# Prevalence and causes of visual impairment in patients seen at Nkhensani Hospital Eye Clinic, South Africa

**DOI:** 10.4102/phcfm.v7i1.728

**Published:** 2015-03-31

**Authors:** Modjadji M. Maake, Olalekan A. Oduntan

**Affiliations:** 1 Department of Public Health, School of Health Sciences, University of Limpopo, South Africa; 2 Department of Optometry, School of Health Sciences, University of Kwazulu-Natal, South Africa

## Abstract

**Background:**

Knowledge of the prevalence and causes of visual impairment (VI) amongst hospital patients is useful in planning preventive programmes and provision of eye-care services for residents in the surrounding communities.

**Aim:**

The aim of this study was to determine the prevalence and causes of VI amongst eye clinic patients at Nkhensani Hospital. The relationship between VI and age was also investigated.

**Setting:**

Nkhensani Hospital in the Greater Giyani subdistrict municipality, Mopani district, Limpopo Province, South Africa.

**Methods:**

Four hundred participants aged 6–92 years were selected for the study using a convenient sampling method. Presenting and best corrected visual acuities (VA) were measured with a LogMAR E chart. Presenting VA (PVA) in the right and left eyes and in the better eye of the patients was used to determine the prevalence of VI, low vision (LV) and blindness. Ophthalmoscope was used to diagnose the eye conditions causing VI amongst participants.

**Results:**

The prevalence of VI based on the PVA in the right eye was 34.8% and in the left eye, the prevalence was 35.8%. There was a significant association between age of the participants and VI in the right and left eyes (*p* = 0.00) in each case, respectively. Based on the vision in the better eye of each patient, the prevalence of VI was 28.0% and there was a significant association between VI and age of the participants (*p* = 0.00). The main causes of VI were uncorrected refractive errors, cataract and glaucoma.

**Conclusion:**

Findings in this study indicate that a large proportion of VI is preventable. Focusing on refractive error correction and surgical intervention for cataract would significantly reduce the burden of VI amongst patients utilising this hospital.

## Abstrait

**Fréquence et causes de déficience visuelle chez les patients examinés à la Clinique phtalmologique de l'Hôpital Nkhensani, Afrique du Sud.**

**Contexte:**

Une connaissance de la fréquence et des causes de déficience visuelle (VI) chez les patients des hôpitaux est utile pour mettre en œuvre des programmes de prévention et des services de soins oculaires pour les habitants des communautés avoisinantes.

**Objectif:**

Le but de cette étude était de déterminer la fréquence et les causes de VI chez les patients de la clinique ophtalmologique de l'Hôpital Nkhensani. On a aussi examiné la relation entre la déficience visuelle et l'âge.

**Cadre:**

L'hôpital Nkhensani dans la municipalité du sous-district du Greater Giyani, district de Mopani, province du Limpopo, Afrique du Sud.

**Méthodes:**

Pour l'étude on a sélectionné quatre cent participants âgés de 6 à 92 ans et utilisé une méthode pratique d'échantillonnage. On a mesuré les acuités visuelles présentes et la meilleure acuité visuelle corrigée (VA) au moyen d'un tableau LogMAR E. On s'est servi de la (PVA) dans l'œil droit et l'œil gauche et dans le meilleur œil des patients pour déterminer la fréquence de VI, la vision basse (LV) et la cécité. On a utilisé un ophtalmoscope pour diagnostiquer les conditions oculaires causant la VI chez les participants.

**Résultats:**

La fréquence de VI basée sur la PVA dans l'œil droit était de 34.8% et dans l'œil gauche elle était de 35.8%. Il y avait une relation significative entre l'âge des participants et la VI dans l’ œil droit et gauche (*p* = 0.00) dans tous les cas, respectivement. En se basant sur la vision du meilleur œil de chaque patient, la fréquence de VI était de 28.0% et il y avait une relation significative entre la VI et l'âge des participants (*p* = 0.00). Les causes principales de VI étaient des erreurs de réfraction non corrigées, la cataracte et le glaucome.

**Conclusion:**

les résultats de cette enquête montrent qu'une grande proportion de VI peut être évitée. En mettant l'accent sur la correction des erreurs de réfraction et les interventions chirurgicales pour la cataracte, on réduirait beaucoup le fardeau de la déficience visuelle chez les patients de cet hôpital.

## Introduction

According to the World Health Organization (WHO),^1^ there are four levels of visual function, namely: normal vision; moderate visual impairment (VI); severe VI; and blindness. Moderate combined with severe VI are grouped together under the term ‘low vision’; and low vision (LV) taken together with blindness represents total visual impairment.^1^ Visual acuity (VA) of less than 6/18 constitutes VI, acuity less than 6/18 to 3/60 constitutes LV and visual acuity less than 3/60 is blindness.^1,2^ From a global perspective, ‘uncorrected refractive errors are the main causes of moderate and severe visual impairment and cataract remains the leading cause of blindness in middle and low income’ countries.^1^ In the past, VI estimates have been based on corrected vision, but in order to assess the magnitude of VI caused by uncorrected refractive errors (URE), estimates need to be based on presenting VA.^2^ In 2010, it was estimated that 285 million people of all age groups were visually impaired, of whom 39 million were blind; the major causes were UREs (43.0%) and cataracts that had not been operated on (33.0%).^3^ The majority of the impairments were correctable, hence preventable.^3^

The prevalence of VI has been reported amongst different populations, with cataracts and refractive errors (RE) being reported as common causes. For example, in a population-based study amongst subjects aged 1–91 years of age in Botucatu, Brazil, Schellini et al.^4^ reported a prevalence of presenting LV (5.2%) and blindness (2.2%) and the main causes were UREs, cataracts and retinal disease. Ramke et al.^5^ found that amongst the people aged ≥ 40 years of age in Timor-Leste, the age, gender and domicile-adjusted prevalence of functional blindness (presenting VA of 6/60 in the better eye) was 7.4% and blindness (≥ 3/60) was 4.1%. The adjusted prevalence of LV (< 6/18 – 6/60) was 17.7%. Cataract was responsible for 72.9% of the cases of blindness and 17.8% of those involving LV. Haq et al.^6^ reported that the prevalence of VI, LV and blindness amongst those members of the population aged 20 years or older in Aligarh, India, based on presenting VA were 13.0%, 7.8% and 5.3%, respectively, whilst the main causes of VI were cataract, RE, glaucoma and corneal opacities. In Tehran, Iran, Fotouhi et al.^7^ found the prevalence of VI to be 2.52% for presenting VA and 1.39% for corrected VA amongst participants aged one year and older. The most frequent cause of VI was UREs (33.6%), followed by cataract (25.4%), macular degeneration (12.7%) and amblyopia (8.2%). Based on the best corrected vision, common causes were cataract (36.0%), macular degeneration (20.0%) and amblyopia (10.7%).^7^

In Nigeria, amongst adults aged ≥ 40 years, Abdull et al.^8^ found that UREs were responsible for 57.1% of moderate VI (< 6/18 – 6/60) and cataract (43.0%) was the most common cause of blindness (VA < 3/60). Cataract-related blindness had a prevalence of 1.8% and glaucoma-related blindness, 0.7%.

In a study of RE and VI amongst school-aged children aged 5–15 years in Durban, South Africa, Naidoo et al.^9^ found that VA of 6/12 or worse in the better eye had a prevalence of 1.4% (uncorrected), 1.2% (presenting) and 0.32% (best-corrected). Refractive errors (63.0%) were the main causes of VI, whilst amblyopia (7.3%), retinal disorders (9.9%), corneal opacities (3.7%), other causes (3.1%) and unexplained causes (12.0%) were responsible for the rest. The main causes of blindness and LV were cataract, posterior segment diseases, glaucoma, uncorrected aphakia and globe abnormalities. Refractive errors (22.0%) were reported as being the cause of LV in their sample population.^9^

Age and gender have an influence on visual impairment and it has been reported that, in all age groups, prevalence increases with age and women have a significantly higher risk of developing VI than men in every region of the world.^3^ This was consistent with the reports by Abdull et al.,^8^ Zainal et al.,^10^ Resnikoff et al.^11^ and Shahriari et al.^12^

Visual impairment has significant socioeconomic implications. Resnikoff et al.^2^ indicated that VI resulting from UREs has both immediate and long term consequences ‘such as lost educational and employment opportunities, lost economic gain for individuals, families and societies, and impaired quality of life’.^2^ In children, poor vision as a result of uncorrected or under-corrected myopia can lead to an inability to read information written on the blackboard and can thus have a serious impact on a child's participation in learning.^13^ This results in poor school performance which will adversely affect a child's educational, occupational and socioeconomic status in life. Visual impairment has also been associated with decreased quality of life (QoL) in persons aged 40 years or older;^14^ correction of RE amongst older people improved their vision-specific QoL.^15^

In a national guideline for the prevention of blindness in South Africa, the Department of Health^16^ reported a 0.75% prevalence of blindness in the country; 80.0% of these cases of blindness were reportedly avoidable. The Department of Health^17^ has also reported a severe lack of epidemiological data on the magnitude of URE in the country. Considering the burden and impact of visually-disabling anomalies on the society and economy, data on their prevalence would be a valuable tool for appropriate planning and resource allocation in the country.

### Aim and objectives

Data on the prevalence and causes of VI in South Africa are few and no studies have been conducted specifically in the Mopani district of Limpopo Province. Hospital data have been used by several authors^18,19,20^ to report eye problems in various populations worldwide, but such a report for South Africa could not be found in the literature. The purpose of this study was to determine the prevalence and causes of VI amongst patients presenting to the eye clinic at Nkhensani Hospital, Limpopo Province. The relationship of VI with age was also examined. Findings reported in this article will provide an insight into the causes of VI amongst patients using the hospital for eye-care services and will be useful for both prevention and intervention planning.

## Research methods and design

### Study setting

Nkhensani Hospital is a level 1 district hospital situated in the Greater Giyani subdistrict municipality, Mopani district, Limpopo Province, South Africa. Most people using Nkhensani Hospital are from the rural areas of the Greater Giyani subdistrict municipality. Eye-care services at the hospital are provided by both optometrists and ophthalmic nurses. Patients who needs specialist care are referred to the ophthalmologist at Elim Hospital or Mankweng Hospital who provides subsequent management and feedback. Where necessary, the diagnosis of the ophthalmologist was used to confirm any ocular diagnosis reported in this study.

### Study population and sampling strategy

The study population was the patients attending the Nkhensani Hospital Eye Clinic in Giyani, Limpopo Province, South Africa between August 2012 and March 2013 – an estimated total population of about 3400 patients. Based on this population size, using the Krejcie and Morgan Table,^21^ a sample of 400 participants was considered adequate for this study. The table provides appropriate sample sizes for listed population sizes, which can be read directly from the table. Resnikoff et al.^11^ found visual impairment to be uniquely distributed across age groups, therefore participants in this study were stratified by age in order to determine the distribution of VI across age strata. Participants were stratified by age (6–18; 19–35; 36–59; ≥ 60 years) and 100 participants were included in each age stratum. All patients six years and older presenting at eye clinic for the first time for eye-care services during the study period were included in the study by the convenient sampling method until the desired number of participants in each age stratum was reached. All those who were recruited agreed to participate in the study. Children below the age of six (possible poor comprehension of instructions) and follow-up patients (to avoid duplication of data) were excluded.

### Data collection

A LogMAR (log of the minimal angle of resolution) illiterate E acuity chart was used to measure presenting (habitual), pinhole and best corrected VA. A pinhole disc was used to detect if reduced VA was a result of RE or eye disease or another anomaly. Where reduced VA resulted from REs, subjective refraction (lenses providing the best vision were determined by the choice made by the patient, when difference lenses were placed in front of their eyes) was done and the REs and corrected vision value recorded. Direct ophthalmoscope examination was used to examine the external and internal structures of the eye. A digital hand-held tonometer was used to measure the intraocular pressure. A confrontation test was performed to estimate the extent of visual field. Those with eye diseases were referred to the ophthalmic nurse and/or ophthalmologist for further management. In cases where the researcher had doubts regarding diagnoses – such as differential diagnoses of the retinopathies – the diagnosis of the ophthalmologist was used to confirm diagnosis. Visual impairment was based on presenting VA and the WHO classification,^2^ modified for LogMAR values using the Holladay^22^ and Johnson^23^ tables. Table 1 below shows the categories and classification of VI used in the study.

**TABLE 1:** Visual acuity ranges, categories and classification of visual impairment according to the World Health Organization classification.

Snellen VA	VA (LogMAR)	Category	Classification
≥ 6/18	0.0 – 0.50	0	Mild or no VI
< 6/18 – 6/60	0.52 – 1.0	1	Moderate VI
< 6/60 – 3/60 (6/120)	1.02 – 1.30	2	Severe VI
< 3/60 – 1/60	1.32 – 1.80	3	Blindness
< 1/60 – LP^†^	1.82 – 3.0	4	Blindness
NLP^†^	4.0	5	Blindness

Note: Moderate and severe visual impairment constitute low vision.

VA, visual acuity; LogMAR, logarithm of the minimum angle of resolution, VI, visual impairment; †, LP is light perception and NLP is no light perception.

### Data analysis

Data were analysed using the descriptive and inferential statistics of the Statistical Package for Social Sciences (SPSS) version 21 (IBM Corp., Armonk, NY 2012). Descriptive statistics (range, mean and standard deviation) were used to describe the cohort and the visual values. The relationship between VI and age was tested for significance using the Chi-squared test; a *p*-value of < 0.05 was considered to be significant at 95% confidence interval.

### Ethical consideration

Approval to conduct the study was obtained from the University of Limpopo Ethics Committee (MEDUNSA), approval number MREC/HS/63/2012:PG. Permission was obtained from the Limpopo provincial department of health, Mopani district Health Office and the Chief Executive Officer of Nkhensani Hospital. Informed consent was obtained from the participants and parents of the children included in the study after they had been provided with appropriate information regarding the purpose and method of the study.

## Results

A total of 400 participants was included in the study, all attending the Nkhensani Hospital Eye Clinic for eye-care services during the period of the study. Their ages ranged from 6 to 92 years, with a mean of 39.5 ± 23.5 years. They comprised 161 (40.3%) men and 239 (59.7%) women.

### Prevalence of visual impairment

The prevalence of VI (combined LV and blindness) based on presenting VA in the right and left eyes (*N*= 400) were 34.8% and 35.8%, respectively (Tables 2 and 3). In the right eye, the prevalence of LV and blindness were 16.3% and 18.5%, respectively; and in the left eye, the prevalence of LV and blindness were 17.5% and 18.3%, respectively. The distribution of the various categories of VI in the right and left eye in relation to the age of the participants is shown in Tables 2 and 3.There was a significant association between age of the participants and VI in the right and left eye (*p*= 0.00). Based on the presenting VA in the better eyes of the patients, the prevalence of VI was 28.0% (LV = 17.1%; blindness = 10.9%) (Table 4). There was a significant association between VI and the age of the participants (*p* = 0.00).

**TABLE 2:** Ages and percentages of participants with various levels of visual status in the right eye based on presenting visual acuity.

Ages (years)	Mild/NVI	Low vision		Blindness			Total VI (%)
	0	1	2	3	4	5	
6–18	22.0	1.5	0.3	0.3	1.0	0.0	3.0
19–35	19.5	2.8	0.0	0.3	1.5	1.0	5.5
36–59	15.0	4.3	0.0	1.5	2.8	1.5	10.1
≥ 60	8.8	7.3	0.3	1.5	6.8	0.5	16.3
**Total**	**65.3**	**15.8**	**0.5**	**3.5**	**12.0**	**3.0**	**34.8**

Note: Mild and no visual impairment (NVI) (category 0), moderate and severe visual impairment (VI) (categories 1 and 2) constituting low vision and blindness (categories 3–5) are shown in the Table. The total percentage of VI participants is shown in the last column.

**TABLE 3:** Ages and percentages of participants with various levels of visual status in the left eye based on presenting visual acuity.

Ages (years)	Mild/NVI	Low vision		Blindness			Total VI (%)
	0	1	2	3	4	5	
6–18	20.8	1.8	0.3	0.8)	1.5	0.0	4.3
19–35	18.8	2.5	0.0	0.8)	1.3	1.8	6.3
36–59	15.5	5.0	0.0	1.0)	2.5	1.0	9.5
≥ 60	9.3	7.8	0.3	1.8)	4.3	1.8	15.8
**Total**	**64.3**	**17.0**	**0.5**	**4.3**	**9.5**	**4.5**	**35.8**

Note: Mild and no visual impairment (NVI) (category 0), moderate and severe visual impairment (VI) (categories 1 and 2) constituting low vision and blindness (categories 3–5) are shown in the Table. The total percentage of VI participants is shown in the last column.

**TABLE 4:** Ages of the participants and percentage distribution of low vision, blindness and visual impairment (VI) based on the visual acuity in the better eye.

Age (years)	Low vision	Blindness	Total VI
6–18	1.5	1.0	2.5
19–35	2.8	1.8	4.6
36–59	4.5	2.8	7.3
≥ 60	8.3	5.3	13.6
**Total**	**17.1**	**10.9**	**28.0**

### Causes of visual impairment

**FIGURE 1:**
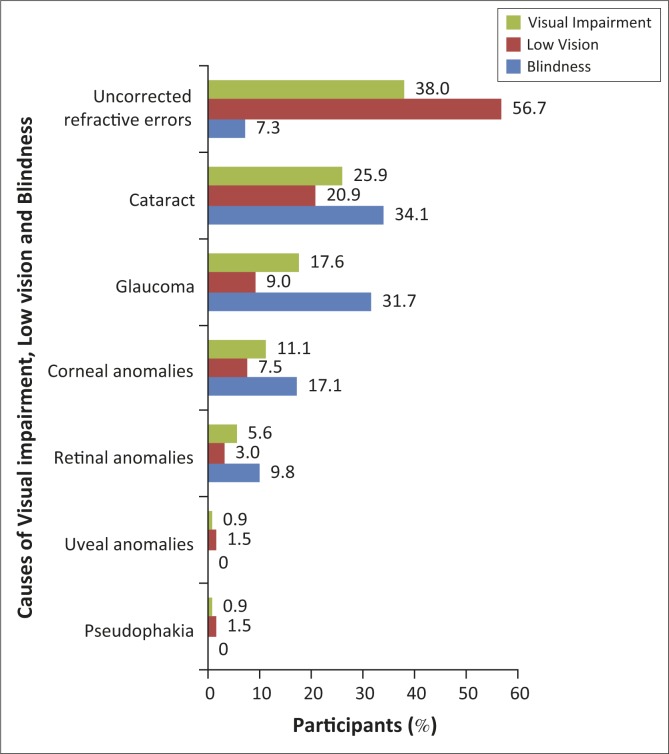
The percentage distributions of causes of visual impairment amongst participants (*N* = 400). Uncorrected refractive errors (UREs) were the most common causes of visual impairment and low vision. Cataract was the main cause of blindness.

The main causes of VI were UREs, cataract and glaucoma (Figure 1) accounting for 38.0%, 25.9% and 17.6%, respectively. The main causes of LV were UREs (56.7%) and cataract (20.9%), whereas the main causes of blindness were cataract, glaucoma and corneal anomalies (accounting for 34.1%, 31.7% and 17.1%, respectively).

## Discussion

Visual impairment is an important public health issue since it impairs the QoL and limits the career choices/job opportunities of those affected, thus constituting a socioeconomic burden on society.^13,14^ It is, therefore, important that the prevalence and causes of the conditions be investigated so that health authorities may have relevant values that can help them in making informed decisions with regard to prevention and management programmes. Population-based studies are the most appropriate method of establishing the prevalence and causes of VI, however, such methods are expensive and time consuming. Hospital-based studies are less expensive and provide useful information that can be used to plan eye-care services in the particular hospital as well as preventive programmes in the surrounding communities. Therefore, this study is of significance in providing data that could be used to improve eye-care services at the Nkhensani Hospital Eye Clinic and may serve as a comparative tool for similar hospital studies in South Africa and other parts of the world.

### Prevalence and causes of visual impairment

The prevalence of VI, LV and blindness were 28.0%, 17.1% and 10.9%, respectively. The main causes of VI were UREs (38.0%), cataract (25.9%) and glaucoma (17.6%). A comparable hospital-based retrospective study^24^ in which the records of all new eye-care patients seen at Adoose Specialist Hospital, Jos, North Central Nigeria were reviewed, found bilateral blindness of 11.0% and LV prevalence of 9.2% in the patients; blindness as well as VI increased significantly with age. Although the prevalence values of LV in that study were lower than found in this study (Tables 2 and 3), the prevalence of bilateral blindness is similar to the 10.9% reported in the present study (Table 4). Refractive errors (33.3%), cataract (28.3%) and glaucoma (13.3%) were also the common causes of VI in that study and their percentages are similar to those found in this study (Figure 1). This similarity reflects the reports in previous population-based studies that REs and cataract,^3,4,5,6,7,8,9^ as well as glaucoma,^8^^9^ are common causes of VI. The findings of UREs and cataract as the main causes of VI in this study are consistent with those in many population-based studies^4,6,7^ and can be attributed to age-related cataracts and to the fact that occurrence of REs is common to all age groups.

Although REs can simply be corrected with a pair of spectacles, the majority of people in South Africa remain visually impaired because of URE. This may be because of an absence of eye-care personnel, poor accessibility to the services or inability to afford the service cost, especially amongst those living in rural and remote areas.

### Relationship between age and visual impairment

The significant association between age and VI (*p* = 0.00) in this study is consistent with that found in various population-based studies.^4,7,8,11^^25,26^ The reason for increase in VI with increasing age, especially amongst the elderly, is a common occurrence in age-related eye conditions such as cataract and glaucoma.

### Limitations

A major limitation of hospital-based studies, including this study, is that they are biased toward those seeking (in this case) eye-care services, hence findings may be higher than would be seen in the population at large. For this reason, the VI prevalence of 28.0% and causes reported here cannot be generalised to the entire district, province or national population. Also, findings should be compared with those in the literature with caution because VI reports in the literature could vary as a result of differences in the ages, study sites or ethnicity of participants as well as the socioeconomic status of the participants. Findings in this study could not be directly compared to the majority of those of the previous prevalence and VI studies because of various factors such as differences in methodology and ages of participants. Also, reports here are based only on presenting, not corrected, VA. Most previous studies on prevalence and causes of VI were population based.^4,5,7,8,9,10,11,12^ Hospital-based studies were few and some of them concentrated only on REs^18,19^ or eye diseases.^20,27^ Furthermore, age differences preclude direct comparison with those studies on REs. For example, the age range of those in the Qureshi et al. study^18^ was 15 to 35 years, hence cannot be compared to the present study where the age range was six to 92 years. Socioeconomic differences may also influence prevalence and causes of VI,^28^ hence should be taken into consideration when comparing these data with other studies.

Although a previous hospital-based study on VI in South Africa could not be found in the literature, findings in this study reflect the views of previous population-based studies in the country which found that cataract^25,29,30^ and REs^9^ are the leading causes of blindness in the country. According to Lecuona and Cook,^29^ ‘human resources available for eye-care and cataract surgery in 2006 in the indigent population are far below the number recommended for the public sector’, hence ‘additional posts for ophthalmologists, optometrists and ophthalmic nurses should be provided and more medical officers trained for cataract surgeries’^30^ We agree with this recommendation because, if implemented, it has the potential to drastically improve eye-care services, reducing the prevalence of cataract and REs and, hence, VI at district, provincial and national levels in South Africa.

### Recommendations

It is recommended that the Department of Health prioritise the elimination of REs and cataract if the prevalence of VI is to be reduced in the country. Sustainable programmes toward correction of REs and cataract surgery are needed in Nkhensani Hospital in order to reduce the burden of VI amongst patients receiving eye-care services in the hospital. As glaucoma is the third most common cause of VI in this study, appropriate programmes should be put in place to detect and manage glaucoma cases before they result in visual impairment. Strengthening awareness programmes and screening campaigns (with appropriate screening equipment) in the Giyani subdistrict where this hospital is located will provide an opportunity for identifying potentially blinding conditions before they cause visual loss.

## Conclusion

This study indicates that the overall prevalence of VI in this hospital sample is high (28.0%), as is shown in Table 4. As the main causes of LV and blindness, based on PVA amongst patients, were UREs and cataract, respectively, VI is preventable as these conditions are correctable. A focus on the optical correction of REs and surgical intervention in the case of cataract would lead to a significant reduction in the burden of VI amongst patients who utilise Nkhensani Hospital for eye-care services.
